# Curcumol alleviates cardiac and cerebral injury caused by encephalomyocarditis virus (EMCV) in Kunming mice via suppressing NF-κB/NLRP3 inflammasome pathway

**DOI:** 10.3389/fphar.2026.1865874

**Published:** 2026-06-30

**Authors:** Panpan Sun, Jiangang Zheng, Abdul Haseeb, Zhenxun Zhang, Na Sun, Yaogui Sun, Hua Zhang, Shaoyu Wang, Hongquan Li

**Affiliations:** 1 Shanxi Key Laboratory for Modernization of TCVM, College of Veterinary Medicine, Shanxi Agricultural University, Taigu, Shanxi, China; 2 School of Public Health, Changzhi Medical College, Changzhi, Shanxi, China; 3 School of Dentistry and Medical Sciences, Faculty of Science and Health, Charles Sturt University, Orange, NSW, Australia

**Keywords:** curcumol, EMCV, Kunming mice, NF-κB, NLRP3

## Abstract

**Introduction:**

Encephalomyocarditis virus (EMCV), a highly significant member of the picornaviridae family, infects a broad range of mammalian hosts and causes severe pathological consequences like encephalitis, myocarditis, and neurologic diseases, etc. In our previous research, curcumol demonstrated a significant reduction in EMCV replication *in vitro*. However, its antiviral effect *in vivo* needs to be elucidated.

**Methods:**

In this study, the mechanism of curcumol against EMCV replication was demonstrated *in vivo* by establishing an EMCV (100 TCID_50_/20 g) infected mice model. At day 3 post EMCV infection, drug treatment groups were continuously treated with curcumol for 5 days.

**Results:**

The viral load and expression of interleukins such as *IL-1β, IL-6,* and *TNF-α* mRNA levels were significantly decreased in the heart and brain tissues at day 9 of EMCV infection (5 days of curcumol treatment), detected by qPCR. Moreover, Western blot analysis revealed that curcumol considerably elevated the protein levels of IκB and P65, while significantly reducing the levels of P-IκB, P-P65, NLRP3 inflammasome, caspase 1, and IL-1β in cardiac and cerebral tissues.

**Discussion:**

Conclusively, these results revealed that curcumol inhibits the NF-κB/NLRP3 inflammasome pathway and thus alleviates heart and brain damage induced by EMCV infection in Kunming mice. The present findings provide new insights into the therapeutic potential of curcumol in addressing EMCV-induced pathological conditions.

## 1 Introduction

Encephalomyocarditis virus (EMCV) is a single-stranded non-enveloped positive-sense RNA virus belonging to the *Picornaviridae* family, genus *Cardiovirus* (Foglia et al., 2023; Li et al., 2025b). EMCV infection results in reproductive abnormalities in sows, acute myocarditis, and sudden death in piglets, and being zoonotic, it can also infect humans, leading to symptoms such as fever and headache (Cardeti et al., 2016; Vansteenkiste et al., 2016; Kim and Moon, 2024; Nederlof et al., 2025). Ge et al. (2010) collected 1786 swine serum samples from 13 provinces and cities that tested positive, with an average seropositivity rate of 75.84%. EMCV infection has broken out on worldwide pig farms in many countries, including Panama, Peru, Italy, South Korea, and Brazil (Jeoung et al., 2015; Gris et al., 2023; Scollo et al., 2023). However, no effective therapeutic drugs and vaccines are available for the prevention and control of EMCV infection (Li et al., 2025a; Yue et al., 2025).

Generally, the host’s innate immune responses determine the severity of EMCV-induced tissue injury. Inflammatory cytokines have been shown to play a significant role in tissue damage (Wang et al., 2025). During this response, activation of Nuclear factor-κB (NF-κB) signaling promotes transcription of proinflammatory cytokines, while assembly of the NOD-like receptor protein 3 (NLRP3) inflammasome drives maturation and release of interleukin IL-1β and IL-18, amplifying local inflammation and cell death. Therefore,. Notably, EMCV proteins and viroporins have been shown to trigger NLRP3 inflammasome activation (for example, via perturbation of intracellular Ca^2+^), linking viral infection directly to inflammasome-mediated pathology in heart and brain. These innate immune pathways, therefore, represent attractive therapeutic targets to limit virus-induced cardiac and neurological damage (Ito et al., 2012; Xu et al., 2023). EMCV’s viral viroporins, particularly EMCV 2B, disrupt intracellular Ca^2+^ homeostasis and serve as a direct activation signal for NLRP3, elucidating the mechanism by which EMCV induces inflammasome-dependent IL-1β production in macrophages and dendritic cells, irrespective of viral RNA detection (Ito et al., 2012).

Furthermore, the EMCV 2B protein induces the efflux of Golgi Ca^2+^ from dendritic cells and macrophages, activates the NLRP3 inflammasome and caspase-1, and facilitates the proteolytic conversion of pro-IL-1β into mature IL-1β. Simultaneously, numerous cardiotropic and neurotropic viruses robustly activate NF-κB signalling in infected tissues; NF-κB facilitates the transcriptional priming necessary for inflammasome responsiveness and stimulates the expression of additional pro-inflammatory mediators that intensify tissue damage in myocarditis and encephalitis (Matsumori, 2023; Xu et al., 2023). Therefore, this tissue damage has been noticed to be reversed in my viral infection, like rotavirus, by inhibiting NF-κB-related pathways (Zheng et al., 2025).

Curcumol is a sesquiterpenoid derived from Curcuma species (*Rhizoma Curcumae*) that has garnered interest for its anti-inflammatory, antioxidant, antiviral, and cardioprotective activities. Recent pharmacological reviews and experimental research indicate that curcumol regulates various signaling pathways associated with inflammation and cell survival, including the inhibition of NF-κB activation and subsequent inflammatory mediators. In addition to NF-κB, recent research has started to delineate curcumol’s impact on upstream innate signaling: comprehensive and mechanistic investigations suggest modulation of AKT pathways, disruption of STING-mediated responses in macrophages, and effects on oxidative stress and mitochondrial dysfunction, each of which can modify NLRP3 activation thresholds in cardiac and cerebral tissues (Yang et al., 2025). In cardiovascular models, curcumol and related Curcuma constituents have been linked to diminished inflammatory responses and mitigation of cardiac remodeling and dysfunction, underscoring their potential to protect the cardiac tissue from injury caused by excessive innate immune activation (Fang et al., 2023; Zhai et al., 2024).

Our previous results showed that curcumol significantly reduced the EMCV load *in vitro* (Zheng et al., 2021). Chen et al. (2014) also showed that curcumol downregulated the NF-κB signaling pathway by inhibiting the degradation of phosphatidylinositol 3-kinase (PI3K) and inhibitor of NF-κB (IκB), thereby playing a therapeutic role in liver fibrosis. Nam et al. (2022) proved that *Curcuma phaeocaulis* inhibits NLRP3 inflammasome in macrophages and ameliorates nanoparticle-induced airway inflammation in mice. Higuchi et al. (2008) found that curcumol reduced the expression of TNF-α and thus alleviated lumbar disc degeneration in mice by activating the PI3K/Akt/NF-kB pathway. However, whether curcumol has anti-EMCV activity *in vivo* and whether it acts on the NF-κB/NLRP3 inflammasome pathway remains unclear. We hypothesized that curcumol could mitigate both cardiac and cerebral damage in EMCV-infected mice through the inhibition of NF-κB signaling and NLRP3 inflammasome assembly. Therefore, the current investigation assessed whether systemic administration of curcumol attenuates EMCV-induced cardiac and cerebral pathology in Kunming mice and examined whether these effects are linked to the downregulation of NF-κB and NLRP3 inflammasome activation.

Previously, our investigation demonstrated that infection with 100 TCID_50_/20 g EMCV resulted in a gradual increase in EMCV viral loads in the heart, brain, and spleen of Kunming mice over time, reaching a peak on the ninth day, with the highest viral loads observed in the heart and brain tissues. The present investigation, therefore, was aimed at examining the effect of curcumol on EMCV activities and on the NF-κB/NLRP3 inflammasome pathway on the ninth day of EMCV infection.

## 2 Materials and methods

### 2.1 Kunming mice, viruses, plasmid, compounds, and antibodies

In total, 48 SPF Kunming mice, 6-8 weeks old, half male and half female (Quality Certificate No.: 1100112101 15155452), were purchased from Beijing Vital River Laboratory Animal Technology Co., Ltd. (SCXK-2021-0006, Beijing). All animal experiments were performed according to the ethical requirements of the Animal Committee of Shanxi Agricultural University (Ethical Review Number: SXAU-EAW-2021 M0111501).

The EMCV NJ08 strain (GenBank: HM641897) was donated by Professor Jiang Ping of Nanjing Agricultural University and was cultured, expanded, and preserved in the laboratory. Its virulence was calculated according to the Reed-Muench method (Chen et al., 2023). The recombinant plasmid for the EMCV *3D* gene was preserved in the laboratory.

Curcumol and ribavirin were purchased from the China Food and Drug Control Institute, with 99.9% and 100% purity, respectively. Curcumol was dissolved in DMEM with 1% DMSO as a cosolvent, while ribavirin was dissolved directly in DMEM before use.

GAPDH mouse monoclonal antibody, β-Actin mouse monoclonal antibody, P65 rabbit polyclonal antibody, P-P65 rabbit polyclonal antibody, IκB rabbit polyclonal antibody, P-IκB rabbit polyclonal antibody, Goat anti-mouse and Goat anti-rabbit secondary antibodies were purchased from Proteintech (United States). Caspase-1 rabbit monoclonal antibody, NLRP3 rabbit monoclonal antibody, IL-1β rabbit polyclonal antibody, and TBP rabbit polyclonal antibody were purchased from Abcam (United States).

### 2.2 Experimental design

Following 1 week of adaptive feeding, all mice were randomly allocated to six different groups, each consisting of 8 mice, including four male and four female mice. The six experimental groups included a control group, a virus group (100 TCID_50_/20 g), and groups receiving high (1.5 mg/kg, High), medium (0.3 mg/kg, Mid), and low (0.06 mg/kg, Low) doses of curcumol, along with ribavirin as a positive control group (40 mg/kg, Rbv). The mice were inoculated with EMCV at day 0 for a duration of 3 days (day 0-3). Following infection, mice received continuous treatment with curcumol for a duration of 5 days (days 4-8). Meanwhile, the control group was intraperitoneally injected with an equivalent volume of normal saline. Mice were monitored daily for clinical signs. On the 9th day of post-EMCV infection, the blood was collected, an autopsy was done, and various organs were harvested for subsequent detection.

### 2.3 qPCR

Total RNA was extracted from the heart and brain tissues of the EMCV-infected mice and curcumol-treated groups according to the Trizol manufacturing protocol (Invitrogen, Carlsbad, CA, United States). The concentration and purity of RNA were evaluated by Eppendorf BioPhotometer D30 (Eppendorf, United States). The complementary DNA was synthesized with Prime-Script® RT Master Mix kit with gDNA Eraser (TaKaRa, Japan) according to the manufacturer’s protocol. qPCR was performed using a 7500 Real Time PCR System (ABI, United States). Relative qPCR was employed to detect the mRNA expression of *IL-1β*, *IL-6,* and *TNF-α* using the 2×SYBR Green qPCR Master Mix (Low ROX, Biotool, United States). Relative expression levels were determined with the 2^−ΔΔCT^ method. Absolute qPCR was applied to determine EMCV *3D* gene level against the standard curve generated using serially diluted plasmid containing the *3D* gene. The primer sequences used for EMCV *3D* gene were: F 5’-TTAGGGCGGGTTTGT AT-3’, R 5’-TTT​GTT​AGC​GGG​AGT​TA-3’. *IL-1β*: F 5’-GCC​ACC​TTT​TGA​CAG​TGA​TGA​GA-3’ R 5’ -GAC​AGC​CCA​GGT​CAA​AGG​TT-3’; *IL-6*: F 5’ -GTC​CTT​CCT​ACC​CCA​ATT​TCC​A-3’ R 5’-TAACG CACTAGGTTTGCCGA-3’; *TNF-α*: F 5’-GAT​CGG​TCC​CCT​TTG​GGA​TG-3’ R 5’-GGTTTGCTACG CAGTGGGC-3’; *β-Actin*: F 5’-CTG​AGC​TGC​GTT​TTA​CAC​CC-3’ R 5’-CGC​CTT​CAC​CGT​TCC​AGT TT-3’.

### 2.4 Histopathological section

The heart apex and left cerebral hemisphere of mice were collected and fixed in 10% formaldehyde solution for 24 h. The tissue samples were then dehydrated with a gradient of low to high concentrations of alcohol and cleared in xylene. The thin tissue sections were immersed in paraffin solution at 60 °C for 2.5 h. After complete paraffin infiltration, the tissue samples were kept in molten paraffin overnight, followed by embedding, and then cooled and allowed to solidify into paraffin blocks. The embedded paraffin block was fixed on a Microtome (Model: RM2255, Leica, Germany) and cut into 5 μm slices. The section was ironed in heated water, pasted on glass slides, and dried in an incubator at 45 °C. Before staining, paraffin was removed from the sections with xylene, and after hematoxylin-eosin (Solarbio, China) staining, the sections were sealed with neutral gum (Solarbio, China), and finally observed by Fluorescence Upright Microscope (Model: DM3000, Leica, Germany).

### 2.5 Western blot analysis

Total protein was isolated from tissues using RIPA lysis buffer. Nuclear and cytoplasmic fractions were prepared with commercial extraction kits (KeyGEN, China). Heart and brain samples were homogenized in RIPA buffer supplemented with 1 mM protease inhibitor and 1 mM phosphatase inhibitor. Protein concentration was determined using the BCA protein assay kit (Beyotime Biotechnology, Jiangsu, China). An equal concentration of all the protein samples was separated on a 10% SDS-polyacrylamide gel, subsequently transferred to a polyvinylidene fluoride (PVDF) membrane. The membrane was then blocked with Tris-buffered Tween 20 (TBST) with 5% non-fat dry milk at 25 °C for 2 h. Next, the membrane was incubated with following primary antibodies overnight at 4 °C: GAPDH and β-Actin mouse monoclonal antibody (1:20000), P65 and P-P65 Rabbit Polyclonal antibody (1:2000), IκB and P-IκB rabbit polyclonal antibody (1:2000), Caspase1 and NLRP3 rabbit monoclonal antibody (1:2000), IL-1β rabbit polyclonal antibody (1:500), TBP rabbit polyclonal antibody (1:5000). Then, the membrane was washed with TBST three times, and incubated with goat anti-mouse and goat anti-rabbit secondary antibodies (1:20000) at 25 °C for 2 h. Finally, the target protein was detected by an enhanced chemiluminescence system (Boster, China). Densitometric values of protein bands were quantified using ImageJ.

## 3 Statistical analysis

All data were presented as Mean ± SD. Data were analyzed using GraphPad Prism^TM^ software 5.0 (GraphPad Software, Inc., California, United States). One-way analysis of variance (ANOVA) followed by a Dunnett’s post-test was used to determine the difference between the groups. Each group consisted of n = 8 (biological repeats), and all the experiments were performed with twice technical repeats. All groups were compared with the EMCV-infected group, *P < 0.05, **P < 0.01, ***P < 0.001.

## 4 Results

### 4.1 Curcumol inhibits EMCV replication in the mice brain and heart

**FIGURE 1:**
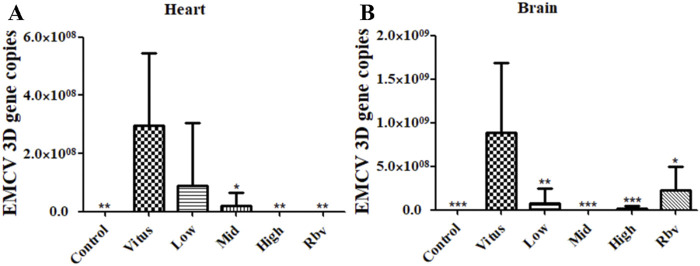
The expression of the EMCV *3D* gene in heart and brain detected by qPCR. **(A)** heart, **(B)** brain (*P < 0.05,**P < 0.01,***P < 0.001).

Post 5 days of curcumol treatment, the EMCV viral loads were quantified in the heart and brain of mice collected from all the groups by qPCR. The results demonstrated that, compared with the virus group, curcumol treatment with low, medium, and high doses significantly (*P* < 0.01) reduced the EMCV viral loads in the heart and brain tissue samples infected with the virus (Figure 1).

### 4.2 Curcumol inhibits heart and brain injury induced by EMCV

**FIGURE 2:**
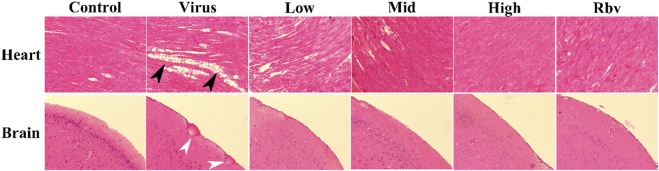
The histopathological sections of heart and brain after 5 days of curcumol treatment (H&E staining, 20 × 10).

The heart and brain tissues were collected at day 5 following curcumol treatment, processed, and stained with H&E. In the control group, the myocardial fibers were clearly visible, evenly and neatly arranged in parallel. The cytoplasm was evenly stained with an oval nucleus in the middle of the cell. Compared with the control group, vacuolar lesions (black arrow) and Submembrane hemorrhage (white arrow) were observed in the cardiomyocytes on the 9th day of EMCV infection. After 5 days of curcumol treatment, the vacuolated lesions and membranous hemorrhage were alleviated, and the myocardial fibers were reverted to the normal condition in comparison with the control group (Figure 2).

In contrast to the control group, characterised by normal meninges and a distinct cortical structure, the EMCV-infected group exhibited dilatation of submeningeal blood vessels, thickened meninges, and submembranous haemorrhage on the 9th day of EMCV infection. Following 5 days of curcumol administration, the submembranous haemorrhages were progressively absorbed, and the submembranous dilated blood vessels were alleviated (Figure 2).

### 4.3 The effect of curcumol on the expression level of *IL-1β*, *IL-6,* and *TNF-α* mRNA

**FIGURE 3:**
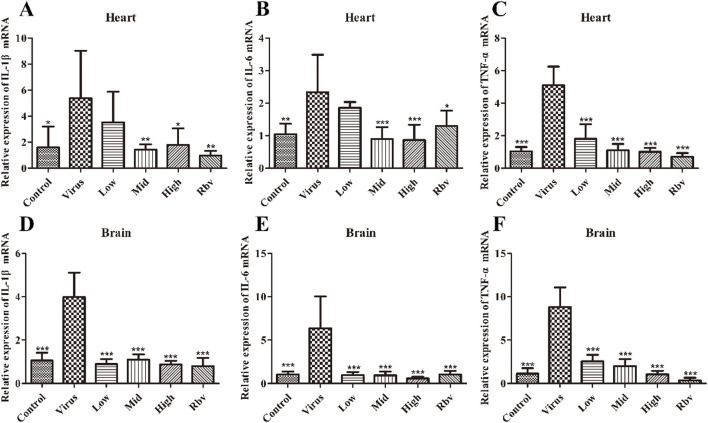
The relative expression level of *IL-1β*, *IL-6,* and *TNF-α* mRNA in the heart and brain detected by qPCR. **(A–C)** Heart, **(C–F)** Brain (*P < 0.05,**P < 0.01,***P < 0.001).

Post 5 days of curcumol treatment, the expression of *IL-1β*, *IL-6,* and *TNF-α* mRNA level in the heart and brain tissues were assessed by qPCR. Compared with the control group, the expression of *IL-1β* (*P* < 0.05), *IL-6* (*P* < 0.01), and *TNF-α* (*P* < 0.001) mRNA levels in the heart of the virus group were significantly increased after 9 days of EMCV infection. Compared with the virus group, post 5 days of treatment, curcumol and ribavirin significantly reduced the expression level of *IL-1β* (*P* < 0.01), *IL-6* (*P* < 0.001), and *TNF-α* (*P* < 0.001) mRNA (Figures 3A–C).

Similarly, compared with the control group, the expressions of *IL-1β* (*P* < 0.001), *IL-6* (*P* < 0.001), and *TNF-α* (*P* < 0.001) mRNA levels in the brain of the virus group were significantly increased after 9 days of EMCV infection. Compared with the virus group, post 5 days of curcumol and ribavirin treatment significantly reduced the expression level of *IL-1β* (*P* < 0.001), *IL-6* (*P* < 0.001), and *TNF-α* (*P* < 0.001) mRNA in the brain (Figures 3D–F).

### 4.4 Expression of NF-κB/NLRP3 inflammasome pathway-related proteins in the heart

**FIGURE 4:**
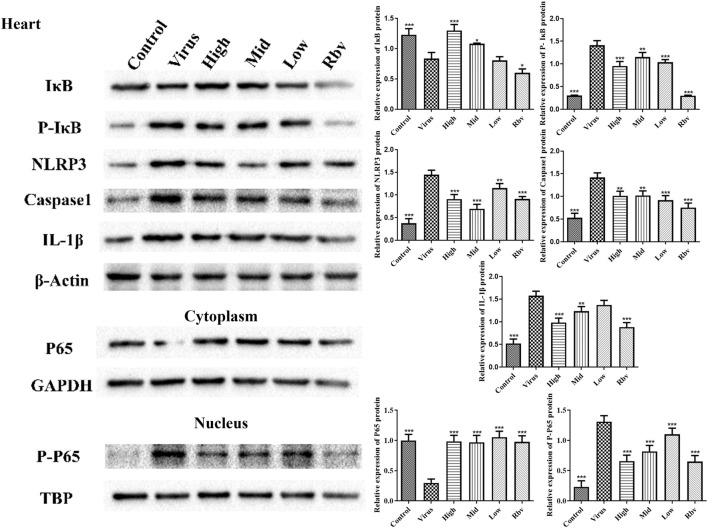
The expression of NF-κB/NLRP3 inflammasome pathway protein in the heart after curcumol treatment. Three concentrations of curcumol (1.5, 0.3, 0.06 mg/kg) and ribavirin (40 mg/kg) were selected to treat EMCV-infected mice, and the expression of IκB, P-IκB, NLRP3, Caspase1, IL-1, P65, and P-65 protein was detected by Western blot (*P < 0.05,**P < 0.01,***P < 0.001).

The expression of NF-κB/NLRP3 inflammasome pathway-related proteins in the heart was detected by Western blot at post 5 days of curcumol treatment. Compared with the control group, the IκB protein and cytoplasmic protein P65 in the virus group were significantly decreased (*P* < 0.001), and the P-IκB, NLRP3, Caspase1, IL-1, and nuclear protein P-P65 were significantly increased (*P* < 0.001). TBP and GAPDH were used as the loading control for the normalization of the expression of P-P65 nuclear protein and P65 cytoplasmic proteins, respectively. These results confirmed that EMCV-infected mice activated the NF-κB/NLRP3 inflammasome pathway-related proteins to regulate the expression of IL-1β. 5 days of curcumol treatment inhibited the NF-κB/NLRP3 signaling pathway and reduced the expression of IL-1β in the heart tissues (Figure 4).

### 4.5 Expression of NF-κB/NLRP3 inflammasome pathway-related proteins in the brain

**FIGURE 5:**
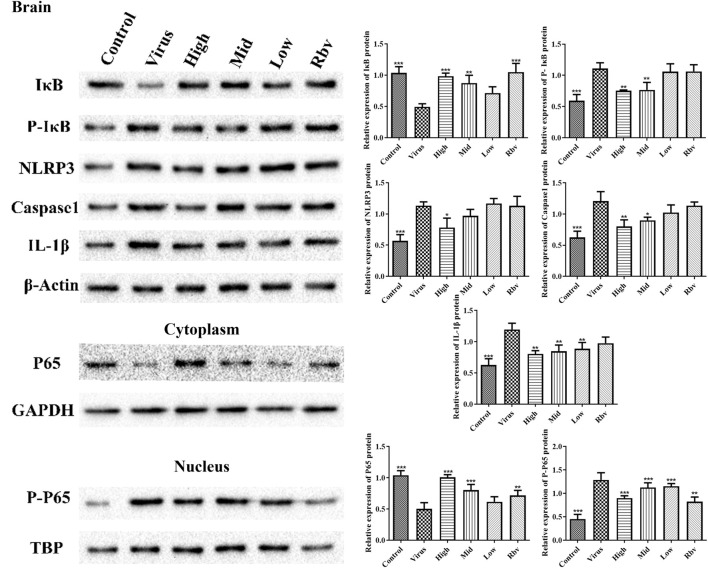
The expression of NF-κB/NLRP3 inflammasome pathway protein in the brain after curcumol treatment. Three concentrations of curcumol (1.5, 0.3, 0.06 mg/kg) and ribavirin (40 mg/kg) were selected to treat EMCV-infected mice, and the expression of IκB, P-IκB, NLRP3, Caspase1, IL-1, P65, and P-65 protein were detected by Western blot (*P < 0.05,**P < 0.01,***P < 0.001).

Post 5 days of curcumol treatment, the expression of NF-κB/NLRP3 inflammasome pathway-related proteins in the brain was detected by Western blot. Compared with the control group, the total cardiac IκB protein and cytoplasmic protein P65 in the virus group were significantly (*P* < 0.001) decreased, and the P-IκB, NLRP3, Caspase1, IL-1β, and nuclear protein P-P65 were significantly (*P* < 0.001) increased. These results confirmed that EMCV activated the NF-κB/NLRP3 inflammasome pathway proteins to promote the expression of IL-1β. After 5 days of curcumol treatment, the NF-κB/NLRP3 signaling pathway was inhibited, and the expression of IL-1β was reduced. The total cardiac protein IκB and cytoplasmic protein P65 tended to increase with the increase in concentration, as exhibited in the control group (Figure 5).

## 5 Discussion

Earlier studies have reported curcumol to have relatively low oral bioavailability (9.2%–13.1%) largely due to its low water solubility and rapid systemic clearance (Yang et al., 2022). However, repeated oral administration of high doses (up to 1,000 mg/kg/day) of curcumol was found to be safe, with no significant toxicity or pathological abnormalities in rats (Yang et al., 2023). The results in the present study indicate that curcumol has a good biosafety profile, while a strategy to enhance its bioavailability may further expand its clinical use. The results showed that curcumol significantly reduced the viral load in the heart and brain post 5 days of curcumol treatment, indicating curcumol’s potent anti-EMCV activity *in vivo*. EMCV-induced myocarditis in mice is characterized by myocardial necrosis and inflammatory cell infiltration, which leads to heart failure and dilated cardiomyopathy (Imanaka-Yoshida, 2020; Nederlof et al., 2025). From the thoracic spine to the lumbar spinal cord, motor neurons that control skeletal muscle activity and tension are also affected by EMCV, and thus lead to paralysis due to the viral acute cytopathic effect on neurons (Brinck Andersen et al., 2021; Khan and Khan, 2021). EMCV may lead to the development of neurological ailments and Neurological manifestations associated with myocarditis (Xie et al., 2024). On the ninth day of EMCV infection, cardiomyocytes exhibited vacuolar lesions, submeningeal vasodilation, meningeal hypertrophy, and submembranous hemorrhage. Following curcumol treatment, myocardial fibers normalized, submembranous hemorrhages were progressively diminished, and submembranous dilated blood vessels were alleviated, indicating the pathological injury mitigation ability of the drug.

Simultaneously, inflammatory factors are considered to play a very important role in the pathogenesis of heart failure (Bartekova et al., 2018). IL-1β reduces cardiac contractility (Abbate et al., 2020), IL-6 leads to negative inotropic force (Liu et al., 2023), and TNF-α causes myocardial contractility disorders, myocarditis, ventricular dilatation, and cardiac hypoplasia (Lutokhina et al., 2025). Certain RNA viruses like VSV and encephalomyocarditis virus (EMCV) activate the NLRP3 inflammasome by an initial signal and subsequent signals to activate an immune response and inflammation (Wan et al., 2022). Studies have shown that, under normal circumstances, EMCV IL-1β expression in the central nervous system is very low, and when the whole body or the local central nervous system is injured and infected in response to several infections, the level of IL-1β significantly increases (Yaseen et al., 2023). Ciryam et al. (2023) found that overexpression of IL-6 in cerebrospinal fluid leads to cytotoxic brain edema and nerve cell damage, and its expression level is positively correlated with the degree of neurological deficit (Stampanoni Bassi et al., 2020). Moreover, IL-6 and TNF-α are considered the most potent proinflammatory cytokines (Haseeb et al., 2025), which act as a novel biomarker for the diagnosis of viral myocarditis (Liu et al., 2022; Zhou et al., 2022). Meanwhile the findings of our study demonstrated that curcumol significantly reduced the mRNA expression levels of *IL-1β*, *IL-6* and *TNF-α* in the heart and brain post 5 days of curcumol treatment, and thus alleviate the myocarditis and encephalitis caused by EMCV infection in mice.

The results revealed that following EMCV infection, IκB and cytoplasmic P65 protein levels in heart and brain tissues were markedly reduced, whereas P-IκB, NLRP3, Caspase1, IL-1β, and nuclear P-P65 levels were significantly elevated, indicating that EMCV infection modulates the NF-κB/NLRP3 inflammasome pathway to promote IL-1β expression. Similarly, macrophage surface C-C motif chemokine receptor 5 (CCR5) recognizes EMCV capsid protein, and thus activates the proinflammatory NF-κB pathway, which in turn stimulates the expression of pro-IL-1β (Shaheen et al., 2015; Xie et al., 2024). CCR5-mediated inflammation contributes to the regulation of EMCV replication and proliferation.

EMCV 2B activates NLRP3 inflammasome and caspase-1, and catalyzes the proteolytic processing of pro-IL-1β into mature IL-1β (Xiao et al., 2019; Choudhury et al., 2021). Curcumol treatment alleviates the inflammatory response of the heart and brain by inhibiting the NF-κB/NLRP3 signaling pathway. (Chen et al., 2014) revealed that curcumol down-regulates the NF-κB signaling pathway by inhibiting the degradation of PI3K and IκB, thereby playing a therapeutic role in liver fibrosis (Nisar et al., 2023; Zhai et al., 2024). He et al. demonstrated that curcumol can reduce the expression of TNF-α and alleviate lumbar disc degeneration in mice by acting on the PI3K/Akt/NF-κB pathway (He et al., 2021). Simultaneously, our findings revealed that curcumol treatment led to significant pathological recovery in both cardiac and cerebral tissues, as demonstrated by substantially less histological damage, reduced inflammatory cell infiltration, and maintenance of tissue architecture compared to untreated, infected controls. Myocardial sections from curcumol-treated groups significantly alleviated vacuolated lesions, and the myocardial fibers were reverted to the normal condition in comparison with the control group. Likewise, brain tissue displayed progressively absorbed submembranous haemorrhages, alleviated submembranous dilated blood vessels, and probable preservation of normal cytoarchitecture. The morphological enhancements were correlated with diminished expression of inflammasome markers (e.g., NLRP3, cleaved caspase-1) and lowered levels of mature pro-inflammatory cytokines like IL-1β, aligning with the inhibition of the conventional inflammasome pathway. The recovery pattern closely resembles cardiac remodeling model induced by adrenergic or pressure-overload stress, curcumol markedly reduced myocardial fibrosis, hypertrophy, and apoptosis, maintained cardiac structure, and enhanced function, indicating its potential to reverse pathological alterations in heart tissue (Fang et al., 2023). Similarly, in central nervous system injury, curcumol has demonstrated the ability to diminish infarct size, neuronal damage, and neuroinflammation following ischemic insult by modulating microglial phenotype and inhibiting NF-κB and oxidative stress pathways (Liu et al., 2024). Therefore, the NF-κB signalling pathway has gained a key significance in antiviral mechanistic response (Yousaf et al., 2025). The histopathological results of this study substantiate the hypothesis that curcumol not only attenuates inflammatory signaling but also facilitates legitimate structural recovery in both the heart and brain following EMCV-induced injury, restoring tissue integrity rather than merely postponing degradation. For clinical translational perspectives, this study highlights the promising therapeutic efficiency of curcumol to overcome pathological injuries. However, there is a further need for pharmacokinetic optimization and validation in large animal models and clinical studies before therapeutic application on a large scale. Moreover, despite its potential therapeutic efficacy against EMCV-induced inflammation, there are some limitations to be considered. To address the deep mechanistic insights of this study, including target discovery, molecular docking, and immunofluorescence analysis of inflammatory cell markers (Qin et al., 2025), can be performed to elucidate the cellular-level influence and target discovery of curcumol. Additionally, experimental approaches, e.g., Cellular Thermal Shift Assay (CETSA) and Microscale Thermophoresis (MST) used in several studies can help in enhancing the mechanistic understanding of the interaction of curcumol with targets (Chen et al., 2026). Future studies employing using these methods will yield more mechanistic information and lead to the clinical translation of curcumol.

## 6 Conclusion

In conclusion, our findings demonstrate that Curcumol exerts robust protective effects against EMCV-induced cardiac and cerebral injury in Kunming mice. These therapeutic advantages are directly associated with its capability to suppress activation of the NF-κB/NLRP3 inflammasome signalling pathway, thereby diminishing the inflammatory responses and mitigating tissue damage. Collectively, this study highlights Curcumol as a potential therapeutic agent for the development of novel antiviral and anti-inflammatory interventions aimed at targeting viral myocarditis and related neurological complications.

## Data Availability

The raw data supporting the conclusions of this article will be made available by the authors, without undue reservation.
